# Comparative Transcriptional Analysis Reveals Differential Gene Expression between Asymmetric and Symmetric Zygotic Divisions in Tobacco

**DOI:** 10.1371/journal.pone.0027120

**Published:** 2011-11-01

**Authors:** Tian-Xiang Hu, Miao Yu, Jie Zhao

**Affiliations:** State Key Laboratory of Hybrid Rice, College of Life Sciences, Wuhan University, Wuhan, China; Duke University Medical Center, United States of America

## Abstract

Asymmetric cell divisions occur widely during many developmental processes in plants. In most angiosperms, the first zygotic cell division is asymmetric resulting in two daughter cells of unequal size and with distinct fates. However, the critical molecular mechanisms regulating this division remain unknown. Previously we showed that treatment of tobacco zygotes with beta-glucosyl Yariv (βGlcY) could dramatically alter the first zygotic asymmetric division to produce symmetric two-celled proembryos. In the present study, we isolated zygotes and two-celled asymmetric proembryos *in vivo* by micromanipulation, and obtained symmetric, two-celled proembryos by *in vitro* cell cultures. Using suppression-subtractive hybridization (SSH) and macroarray analysis differential gene expression between the zygote and the asymmetric and symmetric two-celled proembryos was investigated. After sequencing of the differentially expressed clones, a total of 1610 EST clones representing 685 non-redundant transcripts were obtained. Gene ontology (GO) term analysis revealed that these transcripts include those involved in physiological processes such as response to stimulus, regulation of gene expression, and localization and formation of anatomical structures. A homology search against known genes from *Arabidopsis* indicated that some of the above transcripts are involved in asymmetric cell division and embryogenesis. Quantitative real-time PCR confirmed the up- or down-regulation of the selected candidate transcripts during zygotic division. A few of these transcripts were expressed exclusively in the zygote, or in either type of the two-celled proembryos. Expression analyses of select genes in different tissues and organs also revealed potential roles of these transcripts in fertilization, seed maturation and organ development. The putative roles of few of the identified transcripts in the regulation of zygotic division are discussed. Further functional work on these candidate transcripts will provide important information for understanding asymmetric zygotic division, generation of apical-basal polarity and cell fate decisions during early embryogenesis.

## Introduction

Asymmetric cell division resulting in daughter cells differing in morphology, identify and function is a universal and fundamental mechanism for many developmental processes in angiosperms, and serves an essential role during embryonic and postembryonic development to generate cell diversity [1], [2]. In the classic model plant *Arabidopsis*, asymmetric cell division is involved in many developmental processes as discussed below. During embryogenesis the first zygotic division generates a smaller apical cell, representing the majority of the embryo proper, and a larger basal cell, that provides the suspensor and hypohysis. Subsequent periclinal divisions of proembryo give rise to the epidermis, ground and vascular progenitors, while the hypophysis division produces a lens shaped cell as a progenitor of the quiescent center (QC) [3]. During postembryonic development, stem cells in shoot and root meristem are maintained by signals from their respective organizing center (OC) and QC. These stem cells undergo asymmetric divisions to generate two daughter cells of differing functions, one that remains a stem cell for retaining meristem, and the other daughter cell that goes through a few rounds of division to provide terminally differentiated cells for the formation of tissues and organs [4]. During pollen development, asymmetric division in pollen mitosis I results in a larger vegetative cell and a smaller generative cell. The latter undergoes a second mitotic division and gives rise to two sperm cells that participate in double fertilization [5]. During development of stomata in leaves, asymmetric divisions of meristemoid mother cells generate smaller triangular meristemoid and larger sister cells, and then the meristemoids generally undergo stem cell-like divisions, regenerating a larger sister cell with developmental plasticity and a smaller cell with meristemoid fate. The latter cell eventually differentiates into a guard mother cell to produce the two guard cells of the stomata [6]. Lateral root initiation starts with the specification of pericycle founder cells and their asymmetric division into flanking small cells. After an ordered set of asymmetric divisions, the lateral root meristem initiates, subsequently grows out, and generates the lateral root [7].

Genetic screens have been used to identify genes that are required for specific asymmetric cell divisions during different developmental processes in *Arabidopsis*. These well characterized genes are mainly classified into ligands, receptors, mitogen-activated protein kinases (MPK), and transcription factors in the signaling pathway [6]. In stomatal development, EPIDERMAL PATTERNING FACTORs 1 and 2 (EPF1 and EPF2) encode small secretory peptides that express in stomatal cells and precursors, and control stomatal patterning through regulation of asymmetric cell division. EPF1 activity is dependent on the TOO MANY MOUTHS (TMM) receptor-like protein and ERECTA family receptor kinases, suggesting that EPF1 may provide a positional signal received by these receptors [8], [9]. Leucine-rich repeat receptor-like kinase is an important family in controlling stomatal patterning, with specific family members like ER, ERL1/2 and TMM regulating the specification of stomatal stem cell fate and the differentiation of guard cells [10], [11]. MPK kinases are required for the successive signal transduction processes, and the signals orient spacing division and enforce non-stomatal daughter cell fates in stomatal development [12]–[15]. Moreover, some MPK kinases are reported to play similar roles in controlling the asymmetric division of *Arabidopsis* zygotes, and the MPK, YODA, is involved in extra-embryonic cell fate decision in the basal lineage, implying that a MPK kinase cascade acts as a molecular switch promoting extra-embryonic fate [16]. Transcription factors comprise the largest group of identified genes involved in regulating asymmetric cell divisions. The basic-helix-loop-helix (bHLH) protein family members, SPEECHLESS (SPCH), MUTE, FAMA, SCREAM (SCRM) and SCRM2 determine successive initiation, proliferation, and terminal differentiation of stomatal cell lineages [13], [17]–[19]. The two plant-specific NAC-domain transcription factors, FEZ and SOMBRERO (SMB) regulate the delicately tuned reorientation and timing of cell division in a subset of stem cells [20], [21]. Another group of GRAS family factors, SCARECROW (SCR) and SHORT-ROOT (SHR), regulate the asymmetric cell division in the root to separate the fates of the endodermis and cortex [22]–[25].

With regard to embryogenesis, two members of the WUSCHEL HOMEOBOX (WOX) factor family, WOX2 and WOX8 are located respectively in the apical and basal daughter cells after the asymmetric zygotic division, and the spatially separated WOX transcription factors mediate differential auxin responses and regulate the formation of the main body axis in embryos [26]–[29]. Another important clue in asymmetric cell division is auxin, and many genes have been identified to be involved in this signaling process: the membrane-anchored auxin efflux facilitators PIN 1/7, the Auxin Response Factor (ARF) proteins ARF 5/7/19 and the transcriptional repressors IAA 12/14 [30]–[35]. An essential gene *GNOM* (*GN*), which encodes a GDP/GTP exchange factor for small G proteins, functions as a regulator for polar vesicle transport and is required for the recycling of auxin transport components [36]. A PINOID (PID) Ser/Thr kinase-dependent binary switch controls the polar subcellular localization of the PIN-FORMED (PIN) auxin efflux regulators, and mediates changes in directional auxin flow to create local gradients for the processes of patterning formation [31]. However, the identification of such genes is still limited for elucidating the gene regulatory network of zygotic embryogenesis, and would require a study of global gene expression by transcriptome analysis. A previous work from our laboratory by Qin and Zhao [37] revealed that in the presence of beta-glucosyl Yariv reagent (βGlcY), a synthetic phenylglycoside reagent that specifically binds AGPs, the first zygote division of tobacco is altered from asymmetric to symmetric, producing a symmetric two-celled proembryo. This provides us an excellent experimental system to study zygotic embryogenesis, especially the critical events of the zygote cell division.

Our previous work revealed that the two daughter cells from the first asymmetric division of zygote exhibit different transcriptional profiles [38]. In continuation of these investigations, here we studied in further detail the cell division process in the zygote by comparing transcriptomes of the zygote and the asymmetric, and symmetric two-celled proembryos of tobacco. Tobacco zygotes and asymmetric two-celled proembryos from ovaries were isolated *in vivo*, while the symmetric two-celled proembryos were obtained from zygote cultures supplemented with βGlcY. For cDNA synthesis, a template-switch mechanism to the 5′end of the RNA template during reverse transcription and long-distance PCR (SMART-PCR) was followed to economize the limiting plant material. Suppression-subtractive hybridization (SSH) and macroarray screening were employed successfully to identify ESTs that were up- or down-regulated during the asymmetric and symmetric divisions in zygotes. The ESTs were analyzed further by gene ontology (GO) terms search and comparative studies on the differential gene expression between the zygote asymmetric and symmetric proembryos. Also, the tissue- and organ-specific expression patterns of the identified transcripts were analyzed. The possible relationship between the differential transcript profiles and their physiological impact are discussed. The putative functions of some of the candidate transcripts in embryogenesis and post-embryonic development are also discussed.

## Results

### Isolation of zygotes and asymmetric two-celled proembryos *in vivo*, and symmetric two-celled proembryos from zygote culture

**Figure 1:**
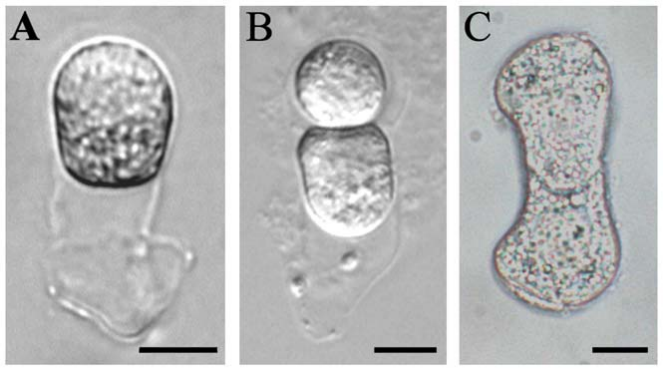
The isolated tobacco zygote and asymmetric two-celled proembryo in *vivo* and symmetric two-celled proembryo *in vitro*. (A) An isolated zygote from ovule at 84 h after pollination; (B) An isolated asymmetric two-celled proembryo from ovule at 108 h after pollination; (C) A symmetric two-celled proembryo from *in vitro* culture for 1–1.5 d. Bar = 10 µm.

**Figure 2:**
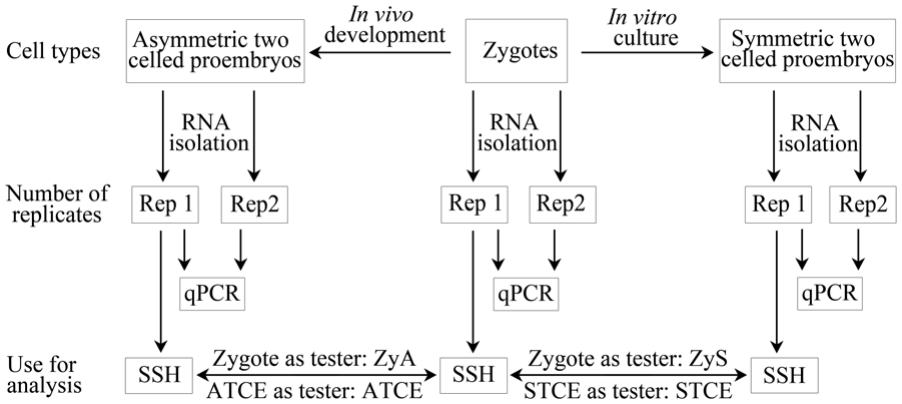
Flow chart describing the material harvest, RNA extraction and the usage for analysis in tobacco. qPCR, real-time quantitative polymerase chain reaction; SSH, suppression subtractive hybridization. ATCE (asymmetric two-celled proembryo), ATCE/zygote subtraction; ZyA, Zygote/ATCE subtraction; STCE (symmetric two-celled proembryo), STCE/zygote subtraction; ZyS, Zygote/STCE subtraction.

In the angiosperm, tobacco, the first zygotic division is asymmetric and produces two unequal-sized daughter cells. By using the well established procedures of micromanipulation combined with enzymatic maceration [37], we isolated the zygotes and two-celled proembryos from ovules at 84 h and 108 h after pollination, respectively (Figure 1A, B). In order to reveal the molecular differences during the asymmetric and symmetric division of zygotes, the symmetric two-celled proembryos were collected from our *in vitro* culture system supplemented with 50 µM βGlcY reagent (Figure 1C), as described earlier [37]. Two independent replicates of each sample were collected, and about 200 zygotes or 150 two-celled proembryos were used for each replicate. As depicted in Figure 2, replicate 1 was used for SSH library construction. To validate the differential expression, both the replicates were used for gene expression analysis by quantitative real-time PCR (qPCR).

### Construction of SSH libraries and identification of differentially expressed genes

**Figure 3:**
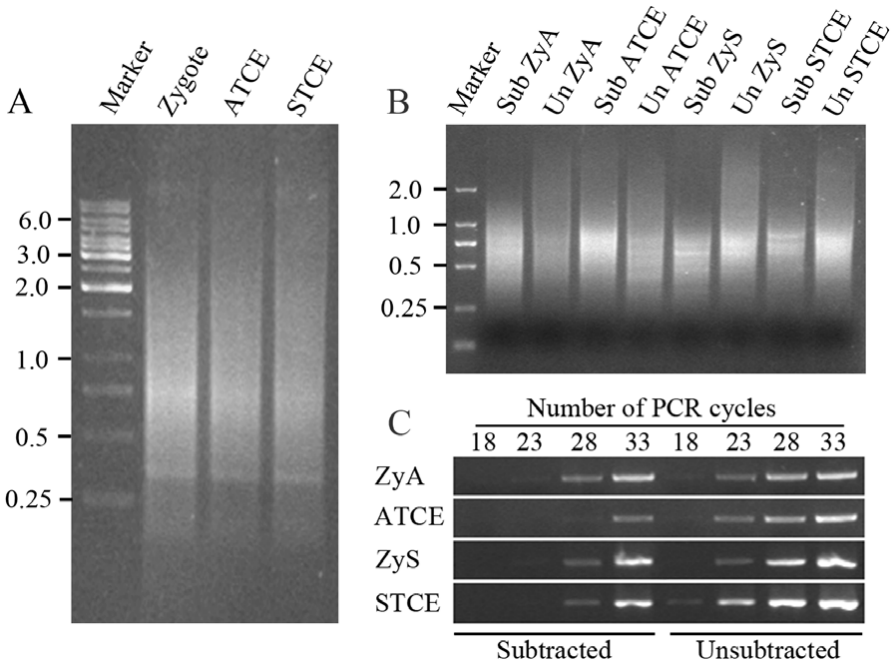
Gel electrophoresis images of cDNA from tobacco zygotes and two-celled proembryos by LD-PCR and products of PCR-select cDNA subtraction, and PCR products for analysis of subtraction efficiency. (A) cDNAs synthesized from the zygotes, asymmetric two-celled proembryos (ATCE) and symmetric two-celled proembryos (STCE). Marker, Molecular marker 1 kb DNA ladder. (B) The second round PCR products of PCR-select cDNA subtraction. Marker, Molecular marker DL2000; Sub ZyA, the product of the zygote/ATCE subtraction; Un ZyA, the product of the unsubtracted control; Sub ATCE, the product of the ATCE/zygote subtraction; Un ATCE, the product of the unsubtracted control; Sub ZyS, the product of the zygote/STCE subtraction; Un ZyS, the product of the unsubtracted control; Sub STCE, the product of the STCE/zygote subtraction; Un STCE, the product of the unsubtracted control. (C) Analysis of subtraction efficiency using PCR. The subtracted and unsubtracted pools of cDNA were amplified by using primers for the housekeeping *GAPC* gene. Aliquots of the samples were taken after 18, 23, 28, and 33 cycles of PCR amplification, and the products were analyzed on a 1.8% agarose gel.

To carry out transcriptional profiling on the limited cell material, the first-strand cDNA was synthesized by applying a template-switch mechanism to the 5′end of the RNA template (SMART) during reverse transcription and then amplified by long-distance PCR (LD-PCR). We optimized the PCR cycle numbers, and carried out individual amplification with 23 PCR cycles to ensure relative equal amplification of the same transcripts in different samples. Generally, the final cDNA products from zygotes and two-celled proembryos ranged from 0.2 to 10 kb, with major abundance between 0.3 and 3 kb as seen by gel electrophoresis (Figure 3A). To reveal the differential transcriptional profiles of zygotes, asymmetric and symmetric two-celled proembryos, the SSH technique was applied. As shown in Figure 2, two forward, asymmetric two-celled proembryo/zygote (ATCE) and symmetric two-celled proembryo/zygote (STCE), and two reverse, zygote/asymmetric two-celled proembryo (ZyA) and zygote/symmetric two-celled proembryo (ZyS) subtracted cDNA libraries were constructed to enrich the up- and down-regulated genes in the asymmetric and symmetric division of zygotes.

Pools of putative differentially expressed cDNA fragments were obtained after two rounds of subtraction. Compared with their respective unsubtracted controls, all the subtracted DNA samples displayed quite a different distribution with a number of distinct bands (Figure 3B). The efficiency of subtraction was also evaluated by PCR amplification of the *GAPC*, a housekeeping gene. If subtraction is efficient, the abundance of housekeeping genes will be greatly reduced, such that the detection of such transcripts by PCR will require more cycles. The results showed that the *GAPC* fragment was clearly detectable in the unsubtracted samples after about 23 cycles of amplification, whereas in the subtracted samples only after 28 or even 33 cycles of amplification (Figure 3C). For equal amplification of the corresponding PCR products in the subtracted and unsubtracted cDNA samples, more than five additional PCR cycles were required, indicating that the differentially expressed genes were efficiently enriched in the subtracted libraries. The resultant subtractive libraries were cloned for differential screening, and the clones with strong differential expression were selected for sequencing.

### EST cluster and comparison analysis

After discarding vector sequences and poor quality sequences, a total of 1610 EST sequences were obtained: 425 originated from the ATCE library (with library ID ATCE001C-425C), 380 from ZyA (with ID ZyA001C-380C), 425 from STCE (with ID STCE001C-425C) and 380 from ZyS (with ID ZyS001C-380C). These isolated sequences were clustered and assembled into contiguous sequences (contigs) and single sequences (singletons) in the four libraries. ATCE consisted of 33 contigs (with ID ATCEC01-33) and 133 singletons, ZyA of 56 contigs (with ID ZyAC01-56) and 107 singletons, STCE of 40 contigs (with ID STCEC01-40) and 179 singletons, and ZyS of 36 contigs (with ID ZySC01-36) and 101 singletons. Thus, these 1610 ESTs represented 685 unique transcripts. Comparison of unigenes in different libraries revealed that only 9 genes were repeated between ZyA and ATCE and 4 genes between ZyS and STCE. The total number of all repeated genes among the 685 transcripts from four libraries was 59, illustrating the high efficiency of the subtraction (Table S1). Results of a BLAST analysis to annotate clusters containing two or more ESTs are shown in Table S2, and the detailed BLAST annotations of all transcripts are listed in Table S3.

In recent years, several different reports have focused on identification of ESTs from egg cells, zygotes and two-cell proembryos, as well as the apical and basal cells in tobacco [38]–[40], providing important information on early embryo development. In the present study, we compared EST transcripts of the four different libraries generated here with previous data (Table S1). Compared with the identified ESTs from zygotes and two-celled proembryos, the ZyA and ZyS, ATCE and STCE libraries add a large number of new transcripts to the current data, with less than 1/3 represented in the previous cDNA libraries (Figure S1). This also proves that the SSH technique is efficient at isolating low abundance and rare expressed ESTs, which is a major advantage over a conventional cDNA library.

### Functional classification of library transcript

**Figure 4:**
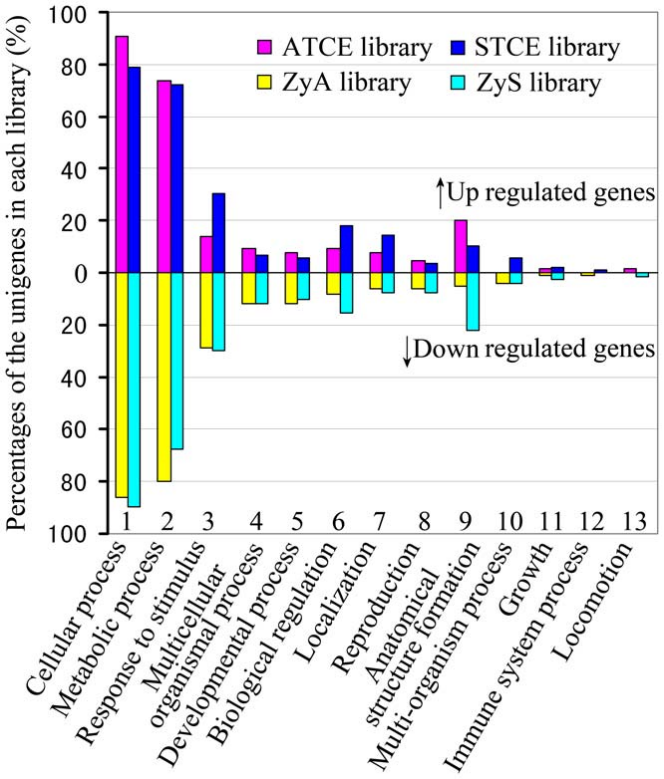
Distributions of differentially expressed unigenes according to the biological process part of Gene Ontology (GO) at 2nd level in the asymmetric and symmetric zygote division of tobacco. The percentages were calculated with respect to unigenes in each library. Since a gene product could be assigned to more than one GO term, the percentages in each main category will add up to >100%.

According to the results of GO mapping, we analyzed the unigene distributions of GO terms (level 2) in the biological processes part of the GO consortium. Figure 4 shows the percentage distribution of unigenes from the zygote and two-celled proembryo libraries. As the developmental process from the zygote to two-celled proembryo is a continuous one, transcripts expressed in the two-celled proembryo were considered as up-regulated after zygote division, while those detected in the zygote were considered as down-regulated during development. During both the asymmetric and symmetric zygotic divisions, transcripts related to cellular and metabolic processes comprised the two major GO categories in all four libraries. Compared with asymmetric division, the up-regulated genes known to be involved in responses to stimuli, regulation and localization were much highly represented in the symmetric division, while those related to anatomical structure formation were poorly represented. Further, among the down-regulated genes, the percentage of genes involved in regulation and anatomical structure formation was much higher in the symmetric division than in the asymmetric division. These results reveal the differential transcriptional alterations between the asymmetric and symmetric zygotic divisions.

### Identification of transcripts related to asymmetric cell division and embryo development

With a view to establishing the identity of the library transcripts generated here, a BLAST homology analysis was performed against previously known genes involved in asymmetric cell division in *Arabidopsis* [1], [2] (Table S4). Among these tobacco transcripts, ZyS038C belongs to the ADP-ribosylation factor (ARF) GTPases family. In *Arabidopsis*, ARF1 is localized to the Golgi apparatus and endocytic organelles, and is involved in the polar localization of PIN-FORMED (PIN) family auxin efflux facilitators to affect apical-basal polarity of epidermal cells [41]. The transcript STCE162C encodes a putative tobacco homolog of the cell wall hydroxyproline-rich glycoprotein ROOT-SHOOT-HYPOCOTYL-DEFECTIVE (RSH) of *Arabidopsis*, which is essential for the correct positioning of the cell plate during cytokinesis in the developing embryo [42]. Another transcript, STCE278C encodes a putative tobacco MPK kinase. In *Arabidopsis*, MAP kinases functions as a key regulator of stomatal development by regulating asymmetric cell divisions and stomatal cell fate specification [12]–[15]. Sequence alignment of these transcripts involved in asymmetric division reveals that they are conserved in many different species (Figure S2 and S3), implying a possibility that these species share similar regulatory mechanisms in cell division and cell fate decision.

Our subtracted cDNA libraries also provide a resource for identifying genes that may be involved in early embryogenesis. Based on the speculation, we listed all embryo-defective (EMB) genes and other embryogenesis involved genes reported in the literature [43]–[46]. Then a BLASTX homology search was performed (with cutoff e-value of ≤10^−5^) to identify tobacco homologs of *Arabidopsis* genes involved in embryo development. A total of 51 transcripts from our four libraries encoded putative homologs involved in embryo development, with similarity to the EMB genes (Table S4). Based on sequence conservation, these tobacco homologs are likely to be involved in embryonic development similar to their *Arabidopsis* counterparts.

### Validation of macroarray transcript profiling by quantitative real-time PCR

**Figure 5:**
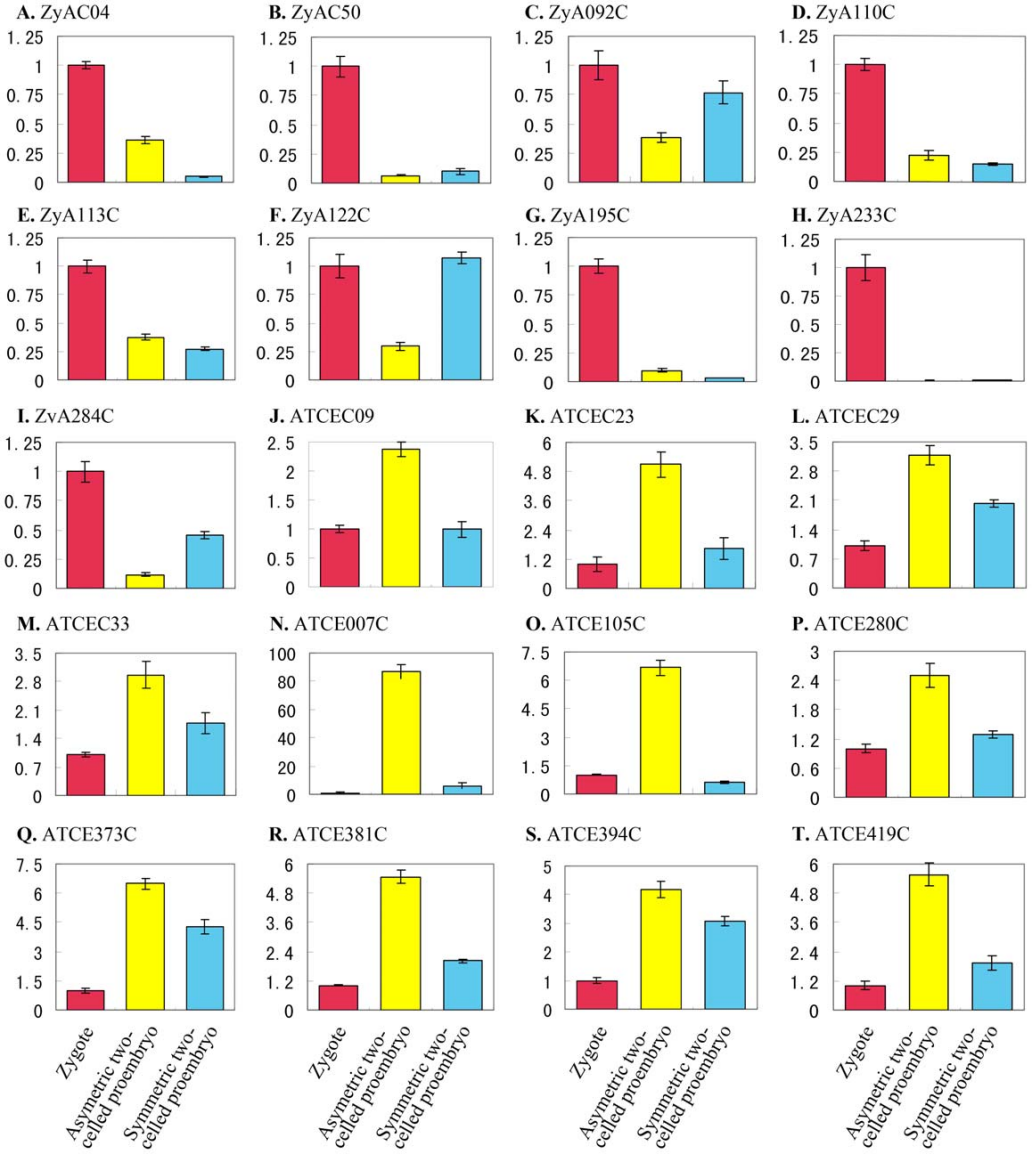
Real-time PCR analysis for transcripts isolated from the asymmetric zygote division in zygotes, asymmetric and symmetric two-celled proembryos of tobacco. The transcripts are indicated by library ID number. The cDNAs synthesized from zygotes, asymmetric and symmetric two-celled proembryos are used as templates for PCR amplification, respectively. The expression level in zygotes is set as reference with the default value 1.

**Figure 6:**
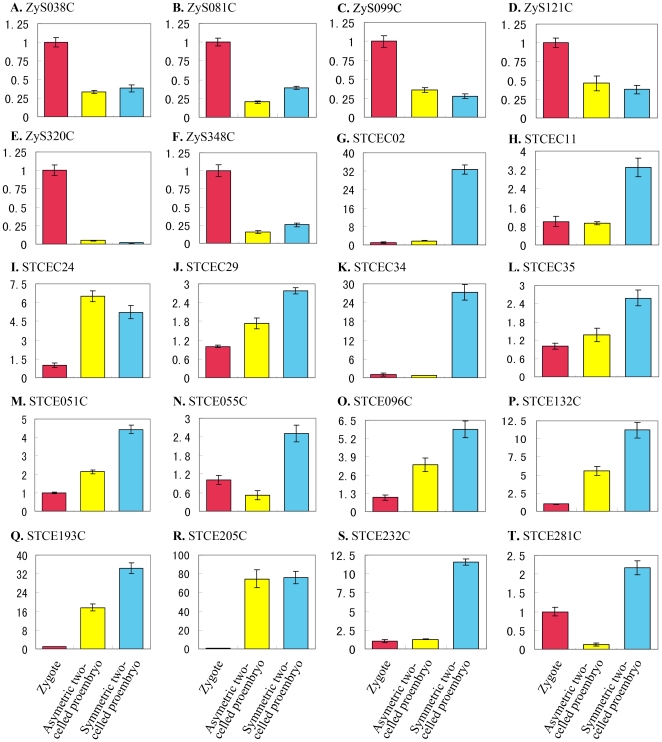
Real-time PCR analysis for transcripts isolated from the symmetric zygote division in zygotes, asymmetric and symmetric two-celled proembryos of tobacco. The transcripts are indicated by library ID number. The cDNAs synthesized from zygotes, asymmetric and symmetric two-celled proembryos are used as templates for PCR amplification, respectively. The expression level in zygotes is set as reference with the default value 1.

Forty differentially expressed genes identified by macroarray analysis were selected for validation of their expression profiles by quantitative RT-PCR with gene-specific primer pairs (Table S5). Transcript abundances were compared in zygote and in asymmetric and symmetric two-celled proembryos (Figure 5 and 6). The cDNA samples from two replicates were used as templates in real-time RT-PCR assay (Figure 2) and each reaction was performed in triplicate. There was no obvious choice of a gene that could serve as an internal control because none of the genes displayed consistent expression among the different samples. Therefore, the expression level of the candidate genes in the zygote was taken as reference with the default value 1, and then the relative expression level in the asymmetric and symmetric two-celled proembryos was calculated. According to this analysis all the forty transcripts tested displayed more than two-fold difference in expression levels during the zygotic asymmetric and symmetric divisions. This confirmed the differential expression of all the selected genes.

The results of real-time RT-PCR show that the differentially expressed transcripts can be classified into six groups based on their general expression patterns: (1) zygote transcripts similarly down-regulated in both the asymmetric and symmetric two-celled proembryos (Figure 5B, D, E, and H; 6A, C, D and E), (2) zygote transcripts down-regulated predominantly in the asymmetric two-celled proembryos (Figure 5C, F and I; 6B and F), (3) zygote transcripts greatly down-regulated in the symmetric two-celled proembryos (Figure 5A and G), (4) transcripts similarly up-regulated in the asymmetric and symmetric two-celled proembryos (Figure 5L, M, Q and S; 6I, J, M, O-R), (5) transcripts highly up-regulated in the asymmetric two-celled proembryos (Figure 5J, K, N-P, R and T), and (6) transcripts up-regulated predominantly in the symmetric two-celled proembryos (Figure 6G, H, K, L, N, S and T). Several transcripts displayed specific expressions, such as ZyAC50, ZyA195C, ZyA233C and ZyS320C in the zygotes (Figure 5B, G and H; 6E), ATCE007C and ATCE105C in the asymmetric two-celled proembryos (Figure 5N and O), and STCEC02, STCEC34 and STCE232C in the symmetric two-celled proembryos (Figure 6G, K and S). The results verified the differential expression of isolated transcripts in the zygote and the asymmetric and symmetric proembryos, and confirmed the utility of our experiment system in identifying zygotic division-related genes.

### Tissue- and organ-specific expression of select candidate genes

**Figure 7:**
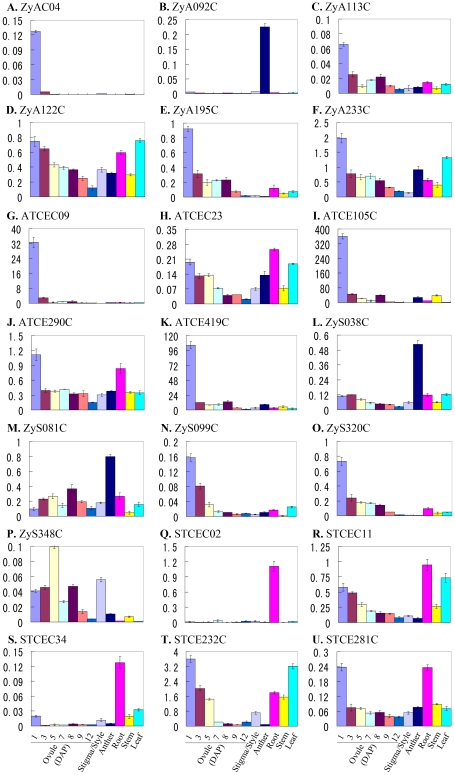
Real-time PCR analysis for transcripts of the four libraries in different tissues and organs of tobacco. All expression presented is relative to that of the reference gene *GAPD*.

The expression of the above validated differentially expressed transcripts in different organs and tissues of tobacco was examined (Figure 7). All of the transcripts displayed differential expression levels in the tested organs and tissues. Most of these transcripts displayed high level expression in ovules at 1 day after pollination (DAP). Among these transcripts, eleven transcripts (ZyA113C, ZyA122C, ZyA195C, ZyA233C, ATCEC23, ATCE290C ZyS099C, ZyS320C, STCEC11, STCE232C and STCE281C) were expressed at high levels in 1 DAP ovules and then the expression levels decreased gradually along with the development of ovules from 1 DAP through 12 DAP (Figure 7 C-F, H, J, N, O, R, T and U). The expression levels of four transcripts (ZyAC04, ATCEC09, ATCE105C and ATCE419C) (Figure 7 A, G, I and K) declined in the ovules immediately after fertilization (at 1 DAP), suggesting their possible roles in ovule development and fertilization. The expression levels of two other transcripts ZyS081C and ZyS348C (Figure 7 M and P) were up-regulated after fertilization and then decreased during seed maturation. Apart from ovules, prominent expression was seen in roots for two transcripts (STCEC02 and STCEC34) (Figure 7 Q and S). Interestingly, the expression of three transcripts, ZyA092C, ZyS038C and ZyS081C (Figure 7 B, L and M) was abundant in anthers but poor in ovules and vegetative tissues. Thus, the results indicate that the isolated transcripts from zygotes and two-celled proembryos may play extensive roles during plant growth and development.

## Discussion

Asymmetric cell division producing two daughter cells with different fates is an important developmental phenomenon that occurs widely from bacteria and fungi to plants and metazoan animals. As a means of generating cell differentiation and diversity, it has attracted widespread attention from researchers in the biology [1], [47], [48]. Embryogenesis, as one of most important events in plant life cycle, is also the phase at which different cells undergo asymmetric division. However, because of the inaccessibility of the deeply embedded zygotes and embryo sacs in the complex sporophytic tissues, investigations on the asymmetric cell division of zygote and early embryos are still rather limited. As a lab focusing on plant sexual reproduction, we have established a technique for isolation of tobacco zygotes and asymmetric two-celled proembryos *in vivo*, and have developed the *in vitro* zygote and proembryo culture system as well. In the *in vitro* culture in a medium lacking βGlcY, most zygotes divided asymmetrically, while the addition of βGlcY results in symmetric cell division [37]. This provides a useful experimental model for studying zygotic cell division and early embryogenesis. Using this model, we carried out an investigation focused on the cell morphologic changes in the processes of zygotic asymmetric and symmetric divisions (unpublished data), and here we present a comparative transcriptional profiling between the zygote and the asymmetric and symmetric two-celled proembryos. Several transcripts displaying differential expression during the asymmetric or symmetric division of zygotes were identified.

As the two-celled proembryo is produced from zygotic division, it implies that the division is complete at the occurrence of the proembryo. Hence a direct comparison of the differential gene expression between the asymmetric and symmetric two-celled proembryos is not meaningful to understand zygotic division *per se*. While compared with the same zygote, isolation of the differentially expressed transcripts between zygote and two-celled proembryos would be useful to understand the regulation of asymmetric and symmetric divisions. The SSH technique applied in this study provides a direct comparison of two given samples, thus we finally compared the differential gene expression between the zygote and the two different types of two-celled proembryos. To verify the results of SSH and macroarray screening, we applied quantitative RT-PCR techniques to examine the expression of candidates. Notwithstanding the difficulty in obtaining the zygote and two-celled proembryo samples, two independent replicate samples were used in quantitative RT-PCR analysis (Figure 2).

### Differential gene expression during the *in vivo* zygote asymmetric and the *in vitro* symmetric zygotic divisions

Once differentially expressed clones from the four SSH libraries were obtained, we compared the constitution of these libraries and tried to identify a general principal or trend (Figure 4). The βGlcY-induced symmetric division serves as a good control to compare the differences in gene expression between asymmetric and symmetric divisions, and then focus on the asymmetric zygote divisions *in vivo*. GO analysis on the biological processes revealed that composition of transcripts related to most biology processes were very similar, and this was also confirmed by real-time PCR results that most of the differentially expressed transcripts in the asymmetric or symmetric division processes also displayed some overlapping expression patterns (Figure 5 and 6). There is a possibility that some of these transcripts related to functions known to be critical in the zygotic division, but were not related to the direct regulation of cell division. Another possibility is that our subtractive libraries represent only a small portion of all the transcripts expressed in the zygotes and two-cell proembryos. The application of microarray or high-throughput sequencing technologies can reveal more information related to these processes.

According to the results of GO analysis, the percentage of differentially expressed transcripts related to anatomical structure formation and localization was vastly different between the asymmetric and symmetric division processes (Figure 4). These transcripts include ATCE160C encoding actin depolymerizing factor 6, STCEC24 and STCE302C encoding alpha tubulin, STCEC29 encoding actin depolymerizing factor 3, STCEC11 encoding cellulose synthase-like protein and STCE081C encoding xyloglucan endotransglucosylase hydrolase protein 9. In BY-2 cells of tobacco and the root epidermis of *Arabidopsis*, treatment with βGlcY resulted in the depolymerization/disorganization of microtubules (MTs) and the formation of thicker actin filaments [49], [50]. In addition, the reagent disturbs the cell plate formation in the protoplast-regenerated cells of *Marchantia polymorpha* [51]. Therefore, identification of the above transcripts may imply that βGlcY treatment induced alteration of cytoskeleton establishment and cell wall position, and further led to the shift of zygotic division from asymmetric to symmetric, indicating that these candidate transcripts may be directly involved in the regulation of asymmetric zygote division.

The βGlcY treatment plays important roles in tobacco zygote symmetric division system *in vitro* [37]. In addition, there were several reports about the complex affects of βGlcY treatment on cell gene expression. In *Arabidopsis* cell suspension cultures, βGlcY treatment induced programmed cell death or wound-like responses, and led to transcriptional modifications most similar to that of wound induction [52]–[53]. In barley aleurone protoplasts, βGlcY disturbed hormone signal transduction, and effectively repressed gibberellin-induced gene expression [54]–[55]. In this study, transcripts in the category of response to stimulus and biological regulation were greatly up-regulated in the zygotic symmetric division (Figure 4). According to our analysis, several tobacco homologs of genes identified by the above reports were isolated in this study, such as STCEC34 encoding WRKY transcription factor and STCE278C encoding the MPK kinase. On one hand, a portion of these genes activated by βGlcY in the symmetric two-celled proembryos possibly participate in the regulation of zygote cell division, and function as primary signal molecules to trigger other response which finally affect zygotic division. One the other hand, it is likely that the βGlcY treatment induced complex variations in transcription of genes besides those involved in symmetric zygote division, therefore, some of our isolated transcripts are not related to the regulation of zygote division, and may be considered as the non-specific effects of βGlcY on zygotic cell division.

### Possible roles of select transcripts in the regulation of asymmetric zygote division

Asymmetric zygotic division is an important event in plant early embryogenesis, and the functional analysis of select genes identified here may facilitate elucidation of the regulatory mechanisms. Although the relation of most of these differentially expressed transcripts with asymmetric cell division is not immediately obvious, BLAST search revealed that some of candidate transcripts maybe involved in cell division regulation. Among these transcripts, ZyA092C encodes a SABRE-like protein. In *Arabidopsis*, SABRE inhibits the activity of ethylene in promoting radial expansion of root cortex cells [56], and the SABRE-like protein encoded by *KINKY POLLEN* (*KIP*) is required for the tip growth of pollen tubes [57]. Besides, the maize *aberrant pollen transmission 1* (*apt1*) gene, a homolog of *SABRE* and *KIP*, was also found to be involved in secretory membrane trafficking for the tip growth of pollen tubes [58]. The transcript ATCEC23 encodes a tobacco homolog of a vacuolar protein sorting (Vps)-associated protein VPS16 from yeast, being a component of the Class C Vps complex. This complex plays essential roles in the processes of membrane docking and fusion at both the transport stages of Golgi-to-endosome and endosome-to-vacuole [59]. Another transcript ZyS038C encodes a protein similar to the ADP-ribosylation factors (ARF) belonging to the ARF GTPase family. The *Arabidopsis* genome includes 6 possible ARFs and their function is essential for vesicle trafficking in the coating and uncoating steps [60]. Among them, the ARF1 is localized to the Golgi apparatus and endocytic organelles, and regulates the apical-basal polarity of root epidermal cells by regulating the localization of the ROP2 and PIN2 proteins [39]. In mouse, the *ARF1* gene plays an important role in regulating asymmetric division of oocyte meiosis, as *ARF1* mutant oocytes undergo symmetric cell division [61]. These identified transcripts are all related to the intercellular trafficking of substance. Hence it is highly likely that they are involved in regulating zygote division by affecting the localization of certain determinants.

Apart from specific cellular localization of certain determinants, cellular signaling transduction participates in the regulation of almost all biology processes. The transcript STCEC34 encodes a putative *WRKY* transcription factor which belongs to a large family of conserved transcription factors. The WRKYs function in pathogen defense, sugar signaling, senescence, trichome development, root growth and phosphate assimilation [62], [63]. A previous microarray analysis revealed that these transcription factors are induced by βGlcY treatment in *Arabidopsis* and barley cells [53], [54]. Moreover, a recent report by Ueda *et al.* [64] revealed that WRKY2 is involved in the regulation of asymmetric zygote division, and links zygote polarization with embryo patterning by the activation of *Arabidopsis* axis patterning genes *WOX8/9*. Another transcript STCE278C encodes a putative tobacco MPK kinase. It was reported that the MPK signaling pathway is activated by βGlcY treatment in *Arabidopsis* cell cultures [53]. The MPK cascades are also known to play important roles in regulating the asymmetric zygote division and the proembryo cell fate decision, and function as key regulators of stomatal development and epidermis patterning [12], [15], [16]. Furthermore, MPK inactivation is discovered in the *ARF1* mutant mouse oocytes, revealing that ARF1 and MPK pathways interact in regulating asymmetric cell division [61].

Auxins have long been proven to play essential roles in the control of cell polarity [30]–[35]. The transcript ZyA233C identified in this study corresponds to a tobacco auxin regulated gene from cultured cells, *arcA*, which encods a G-protein beta subunit-like protein regulated by auxin [65]. Its *Arabidopsis* homolog, *RACK1A*, encodes a protein with similarity to a mammalian receptor for activated C-kinases (RACKs), mediates the signaling responses of gibberellin (GA), abscisic acid (ABA), brassinosteroid (BR) and auxin, and plays regulatory roles in multiple developmental processes including flowering and seed germination [66], [67]. The transcript ZyS081C encodes a protein similar to proton pump interactor 1, which regulates the activity of H^+^-ATPase [68]. In tobacco, our previous work has indicated that the polar distribution and transport of IAA begins from the zygote stage, and plasma membrane (PM) H^+^-ATPase may play a role in zygote and embryo development mediated by IAA signal transduction [69]. Another transcript, STCEC02, encodes the auxin-regulated protein *parA*, which was isolated from a tobacco mesophyll protoplast cDNA library by differential screening [70] and accumulates in leaves in response to *P. solanacearum* infection [71].

In conclusion, the transcripts identified in this study appear to be involved in the regulation of asymmetric zygotic division. Detailed functional studies of candidate genes from the libraries generated here will help elucidate the mechanisms involved in asymmetric zygotic division and cell fate decision in early embryogenesis in tobacco.

## Materials and Methods

### Accession numbers

All 1610 ESTs sequences in the study (library ID ATCE001C-425C, ZyA001C-380C, STCE001C-425C, ZyS001C-380C) were deposited in GenBank with accession numbers from JG448571 to JG450180.

### Isolation of zygotes, asymmetric and symmetric two-celled proembryos

Tobacco (*Nicotiana tabacum* cv. SR1) plants were grown in a greenhouse with a photoperiod of 16 h light/8 h dark at 25–27°C. Zygotes and the asymmetric two-celled proembryos were respectively isolated from ovules at 84 and 108 h after pollination (HAP) according to the method of Qin and Zhao [37]. Two transcription inhibitors, 50mg/L actinomycin D (Sigma) and 100mg/L cordycepin (Sigma), proven to be effective in suppressing the expression of stress-inducible genes [72], were added to all solutions used in the process of isolating cells. The zygotes and two celled embryos were transferred into fresh 13% (w/v) mannitol droplets to be washed twice, then collected respectively into the lysis/binding buffer and immediately frozen in liquid nitrogen. The viability of the isolated cells was detected using 50 mg/L fluorescein diacetate (FDA; Sigma) staining. Zygote culture was carried out as described by Qin and Zhao [37] to obtain symmetric two-celled proembryos, and 50 µM βGlcY reagent (Biosupplies Pty, Australia) was added to the medium. The zygotes were cultured in the dark at 25°C for about 1-1.5 d, and then the symmetric two-celled proembryos was collected.

### RNA isolation, subtractive cDNA library construction and differential screening

The RNA from about two hundreds of zygotes or one hundred and fifty two-celled proembryos was extracted using the Absolutely RNA Nanoprep Kit (Stratagene) according to the manufacturer's instructions. Following DNaseI treatment, the RNA was reversely transcribed and amplified using a Super SMART PCR cDNA Synthesis Kit (Clontech). Then, amplified cDNAs of the zygotes, asymmetric and symmetric two-celled proembryos were used for SSH (suppression subtractive hybridization) and PCR templates for gene-specific expression analysis. Both forward (two-celled proembryos as tester and zygotes as driver) and reverse (zygotes as tester and two-celled proembryos as driver) SSH libraries were constructed using the PCR-Select cDNA Subtraction Kit (Clontech). The abbreviations for these four libraries are ATCE (asymmetric two-celled proembryo), ATCE/zygote subtraction; ZyA, Zygote/ATCE subtraction; STCE (symmetric two-celled proembryo), STCE/zygote subtraction; ZyS, Zygote/STCE subtraction. The library construction and screening procedures were the same as our previous description [38]. In brief, the subtracted zygote, asymmetric and symmetric two-celled proembryo cDNA pools were cloned into *Escherichia coli* DH5α cells. Colony and cDNA macroarrays were used respectively for the first and second round screenings. The probe labeling and macroarray hybridization were carried out using the PCR-select Differential Screening Kit (Clontech) following the user manual. The hybridization signals were recorded by PhosphorImager screens (Amersham Biosciences), and the images were acquired by scanning the membranes with a Typhoon 9210 scanner (Amersham Biosciences). After data analysis using ArrayVision 8.0 software (Amersham Biosciences), the resultant clones showing the most marked differential expressions were picked for sequencing.

### Sequence and bioinformatics analysis

The differentially expressed clones identified by screening were picked for sequence analysis with ABI3730 machines (Applied Biosystems). The vector and adaptor sequences were trimmed using Vector NTI Advance 9 software (Informax). The effective sequences were clustered and assembled into contigs using online tool EGassembler (http://egassembler.hgc.jp/; [73]). The consensus sequence of contigs was used for BLASTN and BLASTX searches using the Blast2GO (http://blast2go.bioinfo.cipf.es/; [74]), with expected value <e^-25^ for BLASTN and an e-value of <e^-5^ for BLASTX. The sequences used for alignment analysis were retrieved from SWISS-PROT database, and sequence alignment was performed by Clustal_X 2.0 software followed by manual adjustment [75], and viewed by the software Jalview [76]. The further gene ontology (GO) data mining was also carried out, and GO terms for cells biological process was analyzed (http://blast2go.bioinfo.cipf.es).

### Differential gene expression validation by quantitative Real-time PCR

For expression analysis in the zygotes, asymmetric and symmetric two-celled proembryo, pre-amplified double-stranded cDNA (ds cDNA) using the Super SMART PCR cDNA synthesis kit was used. After ds cDNA purification and concentration measurement, 20 ng of ds cDNA from each sample was used as template for real-time PCR analysis with SYBR-green fluorescence using the Rotor-Gene Q6000 (Corbett Life Science). Cycling parameters were as follows: 94°C for 10 sec, 56°C for 20 sec, and 72°C for 30 sec by 40 cycles. To minimize experimental error, two independent cDNA replicates for each sample were used and three experiment repeats were carried out. For every examined gene, the expression levels in two-celled proembryos relative to zygotes were calculated. Primer pairs were all designed with Primer Premier Software (Premier Biosoft International) and listed in the Table S5.

### Quantitative Real-time PCR analysis of gene expression in different organs and tissues

For expression pattern analysis among different organs, root, stem and leaf were harvested from the one-month-old plants, and anther and stigma/style from anthesis-stage flowers. Ovules 1 day after pollination (DAP) at the egg-celled stage, 3 DAP at the zygote stage, 5 DAP at early globular embryo stage, 7 DAP at late globular embryo stage, 8 DAP at heart-shaped embryo stage, 9 DAP at torpedo-shaped embryo stage and 12 DAP at cotyledon-staged embryo stage were collected respectively. RNAs were isolated from different organs and tissues using TRIzol reagent (Invitrogen) and from the ovules using Concert Plant RNA Reagent (Invitrogen) according to the manufacturer's protocol. The quality of the total RNA was analyzed by agarose gel electrophoresis. Before RT-PCR, 1 µg of total RNA was digested with DNAseI (RNAse free; Invitrogen) and subsequently used for first-strand cDNA synthesis using SuperScript First-Strand Synthesis System (Invitrogen) according to the manufacturer's protocol. Each reaction contained equal amount of sample cDNA, and was repeated at least twice. The constitutively expressed glyceraldehyde-3-phosphate dehydrogenase (*GAPD*) gene (Accession number AJ133422) was used as an internal standard. Primer pairs were all designed with Primer Premier Software (Premier Biosoft International) and listed in the Table S5.

## Supporting Information

Figure S1 **Distribution of unigenes derived from ZyA, ZyS, ATCE and STCE** **compared to the previous zygote and two-celled proembryo EST clusters.** Unigene comparison between ZyA/ZyS and zygote, ATCE/STCE and two-celled proembryo.
(TIF)

Figure S2 **Sequence alignment of ADP-ribosylation factors (ARFs).** ZyS038C represents the transcript in this work. The others are P18085 from *Homo sapiens*, P36397 and Q9SRC3 from *Arabidopsis thaliana*, P40945 and P61209 from *Drosophila melanogaster*, P49076 from *Zea mays*, P49702 from *Gallus gallus*, P51643 and P51644 from *Xenopus laevis*, P51823 and Q06396 from *Oryza sativa*, P61206, P61751, P84079, P84082 and P84083 from *Rattus norvegicus*, Q10943 from *Caenorhabditis elegans*, Q3SZF2 from *Bos Taurus*, Q61LA8 from *Caenorhabditis briggsae*.
(TIF)

Figure S3 **Sequence alignment of mitogen-activated protein kinases (MPKs).** STCE278C represents the transcript in this work. The others are O23236, Q39021, Q39022, Q39023, Q39024, Q39025, Q39026, Q39027, Q8GYQ5, Q9LMM5, Q9LQQ9 and Q9M1Z5 from *Arabidopsis thaliana*, and Q336X9, Q5J4W4, Q5Z859, Q6Z437, Q84UI5 and Q10N20 from *Oryza sativa*.
(TIF)

Table S1 **Comparison of library transcripts with previous tobacco EST Clusters.**
(XLS)

Table S2 **Functional annotation of the differentially expressed contigs with two or more ESTs in the zygotes, asymmetric and symmetric two-celled proembryos.**
(XLS)

Table S3 **List of library transcripts information according to blast annotation results.**
(XLS)

Table S4 **Putative tobacco homologs of the** ***Arabidopsis*** **genes involved in regulating asymmetric cell division and embryo development.**
(XLS)

Table S5 **Primer pairs for real-time PCR analysis.**
(XLS)
