# Supplementary figures and images for: Comparative Transcriptional Analysis Reveals Differential Gene Expression between Asymmetric and Symmetric Zygotic Divisions in Tobacco
Source: PLoS One. 2011 Nov 1;6(11):e27120. doi: 10.1371/journal.pone.0027120 (PMC3206072; doi:10.1371/journal.pone.0027120)

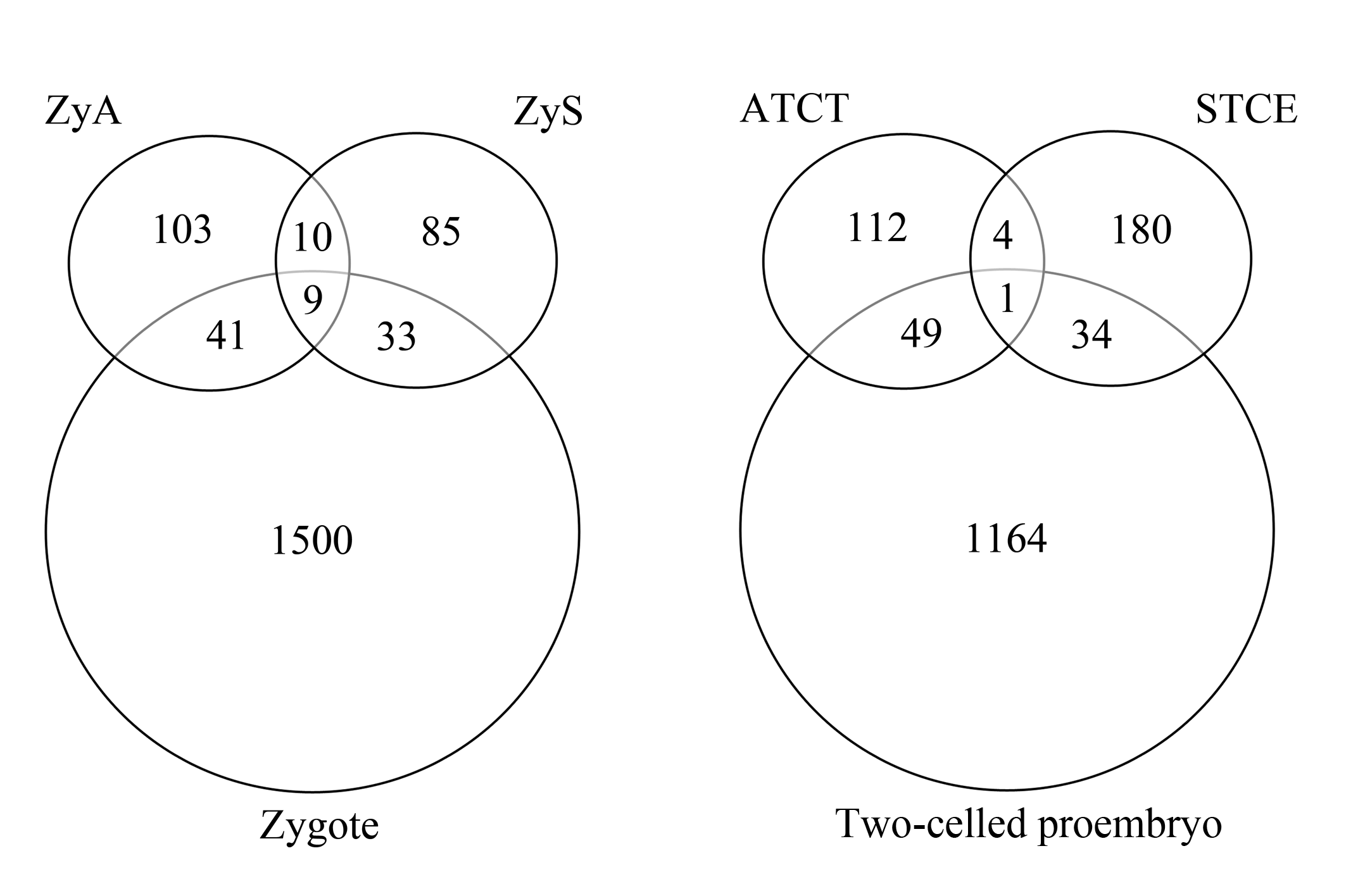

Supplement: Figure S1 — Distribution of unigenes derived from ZyA, ZyS, ATCE and STCE compared to the previous zygote and two-celled proembryo EST clusters. Unigene comparison between ZyA/ZyS and zygote, ATCE/STCE and two-celled proembryo. (TIF) [file pone.0027120.s001.tif]

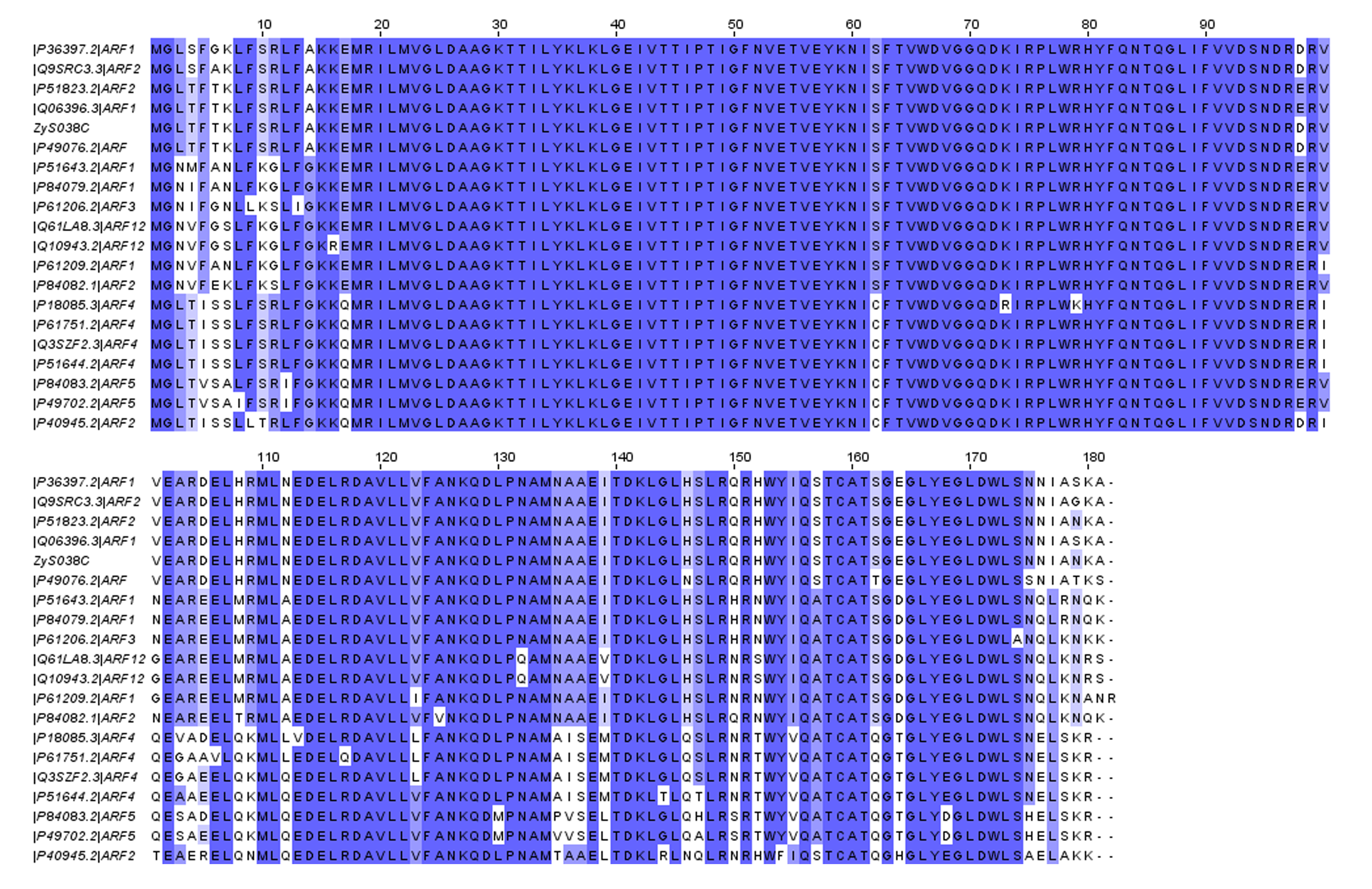

Supplement: Figure S2 — Sequence alignment of ADP-ribosylation factors (ARFs). ZyS038C represents the transcript in this work. The others are P18085 from Homo sapiens, P36397 and Q9SRC3 from Arabidopsis thaliana, P40945 and P61209 from Drosophila melanogaster, P49076 from Zea mays, P49702 from Gallus gallus, P51643 and P51644 from Xenopus laevis, P51823 and Q06396 from Oryza sativa, P61206, P61751, P84079, P84082 and P84083 from Rattus norvegicus, Q10943 from Caenorhabditis elegans, Q3SZF2 from Bos Taurus, Q61LA8 from Caenorhabditis briggsae. (TIF) [file pone.0027120.s002.tif]

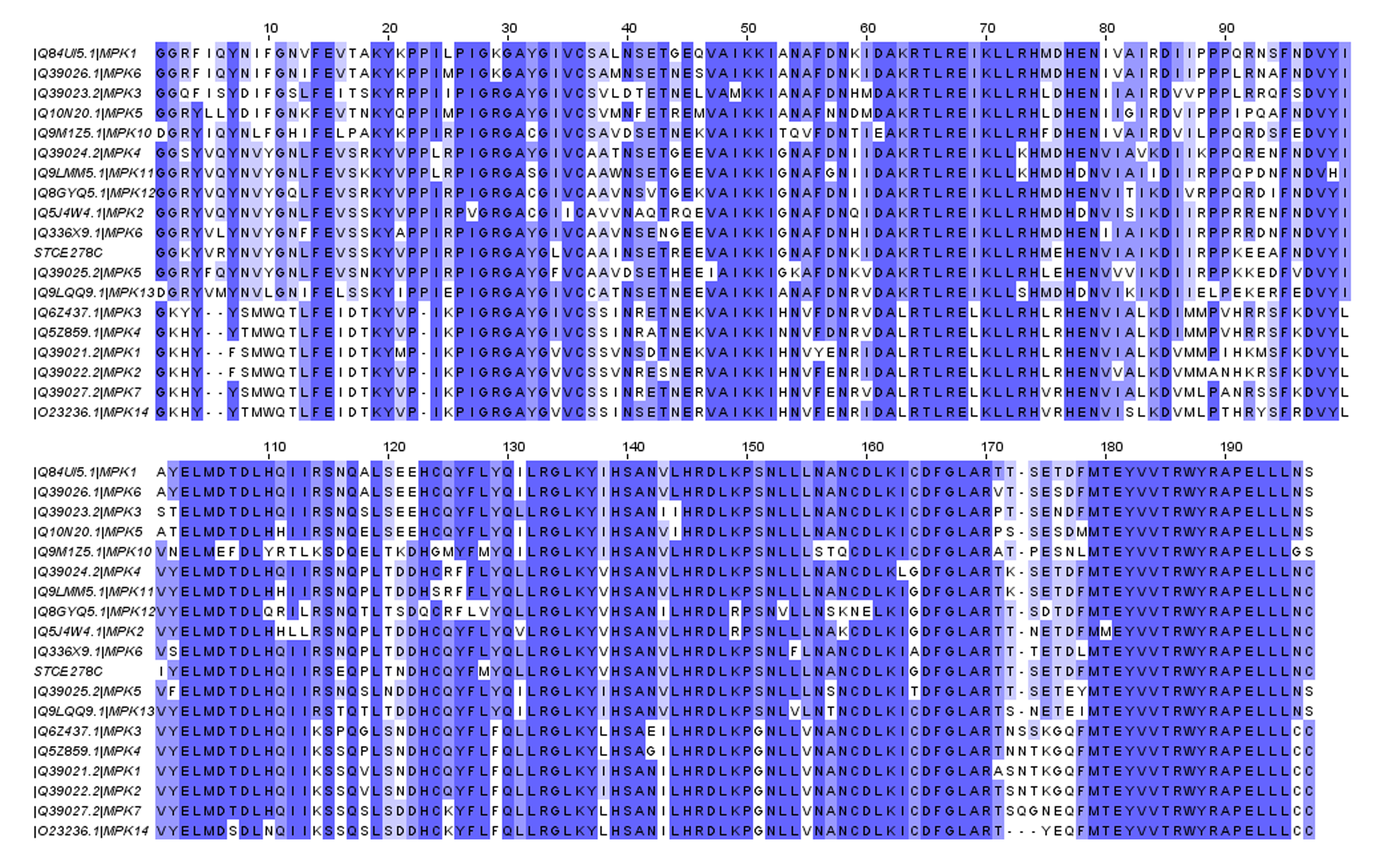

Supplement: Figure S3 — Sequence alignment of mitogen-activated protein kinases (MPKs). STCE278C represents the transcript in this work. The others are O23236, Q39021, Q39022, Q39023, Q39024, Q39025, Q39026, Q39027, Q8GYQ5, Q9LMM5, Q9LQQ9 and Q9M1Z5 from Arabidopsis thaliana, and Q336X9, Q5J4W4, Q5Z859, Q6Z437, Q84UI5 and Q10N20 from Oryza sativa. (TIF) [file pone.0027120.s003.tif]
